# Cardiorespiratory impact of COVID-19 in young adults: A propensity score-weighted cohort study

**DOI:** 10.1016/j.nmni.2025.101598

**Published:** 2025-05-17

**Authors:** Thibault Lovey, Nejla Gültekin, Zeno Stanga, Andreas Stettbacher, Jeremy Werner Deuel, Patricia Schlagenhauf

**Affiliations:** a University of Zürich, Epidemiology, Biostatistics and Prevention Institute, Hirschengraben 84, 8001, Zürich, Switzerland; b Medical Services, Centre of Competence for Military and Disaster Medicine, Swiss Armed Forces, Bern, Switzerland; c University of Zürich, Division of Medical Oncology and Haematology, Zürich, Switzerland; d WHO Collaborating Centre for Travellers' Health, Department of Global and Public Health, MilMedBiol Competence Centre, University of Zürich, Hirschengraben 84, 8001, Zürich, Switzerland

**Keywords:** COVID-19, Post-cute COVID-19 syndrome, Exercise test, Respiratory function tests, Spirometry, Blood pressure, Cardiovascular deconditioning, Sedentary behavior

## Abstract

**Background:**

The COVID-19 pandemic has raised concerns regarding persisting cardiorespiratory effects in young adults. This study aimed to assess the impact of COVID-19 on cardiopulmonary function in this population.

**Methods:**

This investigation, using data from the LoCoMo study, evaluated young recruits from the Swiss Armed Forces, aged 18–30 years. Participants were categorized based on their SARS-CoV-2 infection status and underwent cardiopulmonary exercise testing (CPET), spirometry, and diffusion capacity tests (DLCO). Propensity score weighting adjusted for confounding factors compared key outcomes between the control and COVID-19 groups.

**Finding:**

We evaluated 242 participants in the control group and 240 in the COVID-19 group. The propensity score-weighted analysis showed no significant differences in most CPET and pulmonary outcomes. The COVID-19 group exhibited a significant reduction in systolic blood pressure (SBP) at peak exercise by 7.68 mmHg (p = 0.001), more pronounced in recent cases (<6 months, 14.60 mmHg, p = 0.002) and persisting after infection in non-recent cases (>6 months, 9.07 mmHg, p = <0.001). There was an increase in V'Epeak [% predicted MVV] by 2.92 % in the COVID-19 group, notably in the “non-recent” subgroup who had been infected more than 6 months previously (p = 0.003).

**Interpretation:**

Young adults can exhibit persisting cardiopulmonary effects post-COVID-19, including reduced systolic blood pressure at peak exercise and increased ventilatory response, likely due to deconditioning and muscle weakness. These findings underscore the importance of maintaining physical activity during recovery to mitigate these effects.

## 1 Introduction

The COVID-19 pandemic, caused by the SARS-CoV-2 virus, has globally impacted millions, leading to significant health concerns extending beyond the acute phase of the infection. Among these concerns, the long-term effects on the cardiorespiratory system have emerged as a critical area of study, particularly in young, previously healthy adults. While the acute symptoms of COVID-19 are well-documented, the lingering effects on cardiovascular and respiratory health warrant further exploration. Studies have demonstrated that even post-recovery from COVID-19, individuals may experience persistent abnormalities in lung imaging and function, along with decreased cardiorespiratory fitness and functional capacity [[1], [2], [3]]. These effects have been noted in various young and healthy populations, including children, adolescents, and athletes, suggesting a broad impact [[4], [5], [6], [7]]. Given the prevalence of COVID-19 and its potential to induce significant long-term health alterations, this study aims to assess cardiorespiratory changes post-COVID-19 in previously healthy young adults.

## 2 Methods

### 2.1 Study design and ethical considerations

This investigation, using data from the Long COVID in Military Organisations (LoCoMo) study [8], focuses on the cardiorespiratory sequelae of COVID-19. The LoCoMo cohort study received ethical approval from the Swiss Cantonal Ethics Committee of Zürich (BASEC 2021-00256) and was registered with ClinicalTrials.gov (NCT04942249). The study followed the STROBE (Strengthening the Reporting of Observational Studies in Epidemiology) checklist to ensure transparent reporting of observational research [9,10]. All data used in this analysis—including vaccination status, infection history, and clinical parameters—were collected prospectively on the day of testing between May and November 2021.

### 2.2 Participants

The LoCoMo study was a longitudinal cohort involving young mainly male recruits in the Swiss Armed Forces, aged 18–30 years. Recruitment targeted individuals with positive or negative RT-PCR tests for SARS-CoV-2 during service between March 1, 2020, and December 31, 2020. Participation was voluntary, with informed consent obtained prior to testing. Free transportation to the test center and complimentary meals/refreshments were provided. Documents and consultations were available in German, French, and Italian.

### 2.3 Setting and measurements

Upon arrival, participants underwent a rapid antigen test (COVID-19 Antigen Detection Kit; Zhuhai Lituo Biotechnology, China) to rule out active SARS-CoV-2 infection, followed by a comprehensive medical assessment. This assessment collected demographic and health-related information, including age, sex, height, weight, smoking status, educational background, and vaccination status. Most comorbidities reported were minor conditions such as seasonal allergies or dermatologic issues. Venous blood samples were drawn to measure routine laboratory parameters, such as basic hemogram and chemistry profiles, with all analyses performed at the University Hospital of Zürich.

Participants were categorized based on their SARS-CoV-2 infection status on the day of testing. A prior positive RT-PCR test accompanied by symptoms (fever, cough, or loss of taste/smell) was required for diagnosis. Symptomatic participants were defined by the presence of at least one of these cardinal symptoms. No further stratification by symptom type, severity, or duration was performed. The PCR test date marked the onset of COVID-19. Participants were categorized into recent COVID-19 (positive test within the previous 6 months) and non-recent COVID-19 (positive test more than 6 months prior) groups. The six-month threshold was selected based on prior literature indicating that many post-COVID-19 physiological alterations, particularly cardiorespiratory symptoms and autonomic dysfunction, tend to attenuate within the first 3–6 months after infection. A positive antigen test or symptoms alone were insufficient for diagnosis. Asymptomatic individuals with a positive serological test for anti-nucleocapsid antibodies (Roche Elecsys Anti-SARS-CoV-2; Roche Diagnostics, Switzerland) but without symptoms were classified as asymptomatic. Participants who tested negative for anti-nucleocapsid antibodies and did not meet symptomatic criteria were assigned to the control “non-exposed” group.

The cardiopulmonary exercise test (CPET) was conducted using a CORTEX CPET bicycle ergometer, following a standardized operating procedure (SOP) adapted from European Respiratory Society (ERS) guidelines [[11], [12], [13], [14]]. Participants wore a face mask, heart rate belt, blood pressure cuff, and pulse oximeter. After a 3-min baseline rest and a 3-min equipment familiarization period, a progressive exercise phase began at 20 W (W), with workload automatically increasing. A trained physician monitored key physiological parameters, including blood pressure, oxygen saturation, and perceived exertion (Borg scale). The test concluded at volitional exhaustion (maximal effort), followed by a 3-min recovery assessing post-exercise responses.

Spirometry and DLCO tests were performed by trained technicians following American Thoracic Society (ATS) criteria to assess lung function [15]. Patient demographics, respiratory medications, and lifestyle factors were recorded before testing. Spirometry measured essential lung volumes through maximal flow-volume loop maneuvers, requiring at least three reproducible attempts (maximum eight). The DLCO test, assessing gas exchange efficiency via inhaled carbon monoxide absorption, required at least two reproducible maneuvers. Rigorous sanitation procedures maintained equipment hygiene.

Fractional exhaled nitric oxide (FeNO) levels were measured using the NIOX VERO® instrument, following strict hygiene protocols to prevent cross-contamination. Patients received guided inhalation and exhalation maneuvers with real-time feedback to ensure proper technique. FeNO data was captured directly in REDCap, while data from all tests (CPET, spirometry, DLCO, FeNO) were stored on a dedicated, password-protected drive.

## 3 Variables

The primary interest was the causal effect of COVID-19 on various cardiopulmonary outcomes.

• **For CPET**, the assessed parameters included:
  o Peak oxygen consumption (V’O_2_peak)
  o Oxygen consumption at the anaerobic threshold (V’O_2_ @ AT)
  o Work rate at peak exercise (WR @ Peak Exercise)
  o Work rate at the anaerobic threshold (WR @ AT)
  o Oxygen pulse at peak exercise (V’O_2_/HR @ Peak Exercise)
  o Oxygen pulse at the anaerobic threshold (V’O_2_/HR @ AT)
  o Heart rate at peak exercise (HR @ Peak Exercise)
  o Systolic blood pressure at peak exercise (SBP @ Peak Exercise)
  o Peak minute ventilation (V'Epeak)
  o Peak minute ventilation as a percentage of maximum voluntary ventilation (V'Epeak [% MVV])
  o Oxygen desaturation during exercise (SpO_2_ Desaturation)
  o Ventilatory equivalent for oxygen at peak exercise (V’E/V’O_2_ @ Peak Exercise)
  o Ventilatory equivalent for carbon dioxide at peak exercise (V’E/V’CO_2_ @ Peak Exercise)
  o Respiratory exchange ratio at peak exercise (RER @ Peak Exercise)
• **Spirometry measurements** included:
  o Forced expiratory volume in 1 s as a percentage of vital capacity (FEV_1_ [% VC], Tiffeneau Index)
  o Forced vital capacity as a percentage of vital capacity (FVC [% VC])
  o Residual volume as a percentage of total lung capacity (RV [% TLC])
  o Functional residual capacity (FRC)
  o Maximum mid-expiratory flow (MMEF)
• **For diffusion capacity**, we evaluated:
  o Total lung capacity (TLC)
  o Diffusing capacity of the lungs for carbon monoxide (DLCO)
  o Transfer coefficient for carbon monoxide (KCO)
• **For lung inflammation**, we measured:
  o Fractional exhaled nitric oxide (FeNO)

## 4 Bias

To minimize measurement bias and ensure reliable data, testing procedures were standardized using the same machines and technicians throughout the study. Only participants who achieved maximal effort during the CPET test (per ERS guidelines) and had spirometry and DLCO data meeting the 2019 ATS criteria were included for analysis [11,15].

Confounding bias was addressed through propensity score weighting. Relevant adjustment variables for estimating propensity scores were identified after a comprehensive literature review [[16], [17], [18], [19], [20], [21]]. Weighting was preferred over matching due to limited sample sizes in certain subgroups. A sensitivity analysis evaluated the robustness of our average treatment effect (ATE) under various assumptions about unmeasured confounding, exploring how hypothetical confounders could influence the observed effects.

### 4.1 Study size

A target sample size of 500 was determined through a priori power calculation, with 21 % of the 2500 contacted participants agreeing to participate. This sample size allows detection of differences amounting to 30 % of the standard deviation (Cohen's d = 0.3), corresponding to a small-to-moderate effect size, with a significance level of 0.05 and a power of 80 %, assuming a normal distribution of the test variable. This threshold was chosen a priori to detect subtle but potentially meaningful physiological changes in a healthy young population.

### 4.2 Statistical methods

Sociodemographic variables and laboratory results were compared between the COVID-19 and control groups, further stratified into subgroups: Control (non-exposed), COVID-19 (asymptomatic), COVID-19 (<6 months), and COVID-19 (>6 months) (See Table 1 for a list of sociodemographic variables.). Continuous variables were summarized as mean, standard deviation, and range, while categorical variables were presented as frequencies and percentages. Missing data for each variable across all subgroups were also displayed.

**Table 1:** Sociodemographic and Clinical Characteristics of Study Participants This table presents the characteristics of study participants, including demographics (age, sex, ethnicity), lifestyle factors (smoking status, physical activity levels, alcohol consumption), health metrics (vaccination status, BMI, asthma prevalence, daily medication usage, comorbidities), and laboratory parameters (CRP, troponin T, NT-proBNP, HbA1c, cholesterol levels, hemoglobin). Comparisons are made between the control and COVID-19 groups, with further subgroup analysis for asymptomatic, recent, and non-recent COVID-19 cases. table_1 docx

Characteristic			*COVID-19 SUBGROUPS*		
	Control, N = 242^a^	COVID-19, N = 240^a^	COVID-19 (asymptomatic), N = 46^a^	COVID-19 (<6 months), N = 19^a^	COVID-19 (>6 months), N = 175^a^
**Age [years]**					
*Mean (SD);Minimum-Maximum*	21.58 (1.54); 19.00–29.00	22.30 (2.41); 19.00–29.00	21.74 (1.97); 19.00–28.00	21.16 (0.83); 20.00–23.00	22.57 (2.57); 19.00–29.00
*Missing*	2	0	0	0	0
**Sex**					
*Female*	13 (5.4 %)	16 (6.7 %)	4 (8.7 %)	1 (5.3 %)	11 (6.3 %)
*Male*	229 (95 %)	224 (93 %)	42 (91 %)	18 (95 %)	164 (94 %)
**Ethnicity**^b^					
*African*	0 (0 %)	3 (1.7 %)	0 (0 %)	0 (0 %)	3 (2.4 %)
*Caucasian*	170 (99 %)	170 (98 %)	35 (100 %)	12 (100 %)	123 (97 %)
*South East Asian*	1 (0.6 %)	1 (0.6 %)	0 (0 %)	0 (0 %)	1 (0.8 %)
*Missing*	71	66	11	7	48
**Smoking status**					
*Never smoked*	126 (52 %)	128 (53 %)	27 (59 %)	9 (47 %)	92 (53 %)
*Former smoker*	33 (14 %)	36 (15 %)	5 (11 %)	1 (5.3 %)	30 (17 %)
*Current smoker*	83 (34 %)	76 (32 %)	14 (30 %)	9 (47 %)	53 (30 %)
**Physical Activities**^c^					
*Insufficiently Active*	80 (33 %)	84 (35 %)	9 (20 %)	4 (21 %)	71 (41 %)
*Moderately Active*	70 (29 %)	67 (28 %)	18 (39 %)	7 (37 %)	42 (24 %)
*Highly Active*	92 (38 %)	89 (37 %)	19 (41 %)	8 (42 %)	62 (35 %)
**Alcohol Consumption**					
*Yes*	141 (58 %)	121 (52 %)	30 (65 %)	8 (42 %)	83 (49 %)
*Occasionally*	83 (34 %)	93 (40 %)	11 (24 %)	11 (58 %)	71 (42 %)
*No*	18 (7.4 %)	20 (8.5 %)	5 (11 %)	0 (0 %)	15 (8.9 %)
*Missing*	0	6	0	0	6
**Vaccinated**^d^	184 (76 %)	157 (65 %)	30 (65 %)	10 (53 %)	117 (67 %)
**BMI [kg/m2]**					
*Mean (SD);Minimum-Maximum*	23.7 (3.3); 17.0–39.2	24.1 (3.6); 17.5–42.5	23.9 (4.4); 17.5–42.5	22.9 (2.8); 19.8–29.9	24.3 (3.5); 19.0–39.3
*Missing*	1	0	0	0	0
**Asthmatic**	16 (6.6 %)	13 (5.4 %)	4 (8.7 %)	1 (5.3 %)	8 (4.6 %)
**Daily Medications**^e^	28 (12 %)	24 (10 %)	4 (8.7 %)	1 (5.3 %)	19 (11 %)
**Comorbidities**^f^	50 (21 %)	44 (18 %)	8 (17 %)	3 (16 %)	33 (19 %)

a n (%).

b Ethnicity data was sourced from CPET records as it was not consistently recorded during medical anamnesis, leading to instances of missing data.

c Classification adheres to the most recent World Health Organization (WHO) guidelines.

d Includes participants who have received at least one dose of a COVID-19 vaccine.

e Includes participants who took medication on the day of the test or who were on a daily medication regimen.

f Encompasses participants with chronic conditions, including mild conditions such as allergies.

The proportion of participants reaching maximal efforts in the CPET and meeting ATS criteria for spirometry and DLCO was reported, along with explanations for missing data. Univariate analysis was performed to compare key outcomes between the control group and both the main COVID-19 group and its subgroups. T-tests or Wilcoxon tests, depending on variable distribution, were used for primary comparisons. Tukey's Honest Significant Difference test was applied to adjust for multiple testing in subgroup comparisons.

Propensity score weighting was employed to adjust for confounding factors and estimate the average treatment effect (ATE). Propensity scores were derived from a logistic regression model incorporating sociodemographic variables (age, sex, ethnicity, smoking status, physical activities, alcohol consumption, BMI, asthma, daily medications, comorbidities) selected based on known associations with the treatment and outcome.

Multiple imputation was utilized to address missing data, generating fifteen complete datasets. Covariate balance across treatment groups was assessed by evaluating standardized mean differences and Kolmogorov-Smirnov statistics before and after applying weights.

A sensitivity analysis was conducted to test the robustness of significant findings against varying magnitudes of unmeasured confounding, systematically varying effect sizes of hypothetical confounders to determine the influence required to negate observed treatment effects.

A significance level of 0.05 was used for all statistical tests. All analyses and data processing were performed using the statistical software R version 4.4.0 (2024-06-14). The R code is available in Appendix A.

## 5 Results

### 5.1 Sociodemographic characteristics

A total of 484 young adults participated in the study, with 242 in the control group and 240 in the COVID-19 group. The COVID-19 group had 46 asymptomatic cases, 19 recent cases (<6 months), and 175 non-recent cases (>6 months). Both groups had an average age of 22 years and were predominantly male; females constituted 5.4 % of the control group and 6.6 % of the COVID-19 group. Most participants were physically active, with one-third being insufficiently active (defined as being physically active once a week or less). Smoking was reported by 34 % of the control group and 32 % of the COVID-19 group. Alcohol consumption was reported by 92 % of both groups. The mean Body Mass Index (BMI) was 24.1 (SD = 3.6) for the COVID-19 group and 23.7 (SD = 3.3) for the control group. The prevalence of asthma, daily medication usage, and comorbidities, including allergies, were similar between the groups. Vaccination rates were 76 % for the control group and 66 % for the COVID-19 group. It should be noted that COVID-19 vaccination for this age group was delayed due to prioritization of high-risk groups in Switzerland. Ethnicity data were inconsistently recorded, leading to missing information partially mitigated by data from CPET records. (Table 1).

### 5.2 Data quality

Due to technical reasons, five CPET results and two diffusion capacity tests were unavailable. However, 99 % of participants in each group achieved maximal effort during CPET per European Respiratory Society guidelines. Specifically, a plateau in V’O2 was observed in 83 % of the control group and 88 % of the COVID-19 group. Peak heart rate (HR) exceeding 100 % of the predicted value was noted in 73 % of controls and 81 % of COVID-19 participants. V'Epeak of at least 85 % of maximal voluntary ventilation (MVV) was achieved by 34 % of the control group and 40 % of the COVID-19 group. The respiratory exchange ratio (RER) surpassed 1.15 in 89 % of the control group and 87 % of the COVID-19 group. Compliance with ATS spirometry criteria was 96 % in both groups. For DLCO, 88 % of the control group and 87 % of the COVID-19 group adhered to ATS standards (Table 2).

**Table 2:** Compliance with Cardiopulmonary Exercise Testing (CPET) and Pulmonary Function Criteria This table shows the compliance of participants with CPET, spirometry, and DLCO criteria, as well as adherence to ERS and ATS guidelines. Comparisons are made between the control and COVID-19 groups, with further subgroup analysis for asymptomatic, recent, and non-recentCOVID-19 cases.table_2 docx

Characteristic			*COVID-19 SUBGROUPS*		
	Control, N = 242^a^	COVID-19, N = 240^a^	COVID-19 (asymptomatic), N = 46^a^	COVID-19 (<6 months), N = 19^a^	COVID-19 (>6 months), N = 175^a^
**CPET**					
Reached Maximal Effort (at least one of):^b^	238 (99 %)	235 (99 %)	46 (100 %)	19 (100 %)	170 (99 %)
*Plateau in V'O2*	200 (83 %)	208 (88 %)	42 (91 %)	18 (95 %)	148 (86 %)
*Peak HR* > *100 % Predicted*	176 (73 %)	192 (81 %)	37 (80 %)	14 (74 %)	141 (82 %)
*V'Epeak* ≥ *85 % MVV*	81 (34 %)	94 (40 %)	14 (30 %)	8 (42 %)	72 (42 %)
*RER* > *1.15*^c^	213 (89 %)	206 (87 %)	39 (85 %)	14 (74 %)	153 (89 %)
*Missing*^d^	2	3	0	0	3
**Spirometry/Diffusion Capacity (DLCO)**					
*Spirometry Criteria*^e^	233 (96 %)	231 (96 %)	43 (93 %)	17 (89 %)	171 (98 %)
*DLCO Criteria*^e^	211 (88 %)	209 (87 %)	43 (93 %)	17 (89 %)	149 (86 %)
*Missing*^f^	1	1	0	0	1

a n (%).

b Criteria are based on the latest guidelines (2019) established by the European Respiratory Society (ERS).

c Adapted in accordance with the latest recommendations.

d Data from one participant on study days — 2nd and 28th of June, 2nd of August, 21st of September, and November 9, 2022 — were unavailable and are presumed to have been missing at random (MAR).

e Criteria for the acceptability, usability, and repeatability of spirometry and diffusion capacity (DLCO) measurements adhere to the 2019 guidelines established by the American Thoracic Society (ATS).

f Measurement was not possible due to technical issues with the CO gas tank.

### 5.3 Mean differences and abnormal proportions

Analysis of aerobic and anaerobic responses showed a lower mean workload at anaerobic threshold (WR @ AT) in the COVID-19 group compared to controls (27 vs. 28, p = 0.040∗), with more pronounced differences in non-recent cases (p = 0.026∗). Despite lower mean values, fewer COVID-19 patients showed abnormal V’O2peak values compared to controls (15 % vs. 22 %, p = 0.045∗). Ventilatory efficiency at the anaerobic threshold (V’O2/HR @ AT) favored controls with higher mean values (59 vs. 58, p = 0.023∗), continuing in non-recent COVID-19 cases (p = 0.025∗). Systolic blood pressure at peak exercise was lower in the COVID-19 group (178 vs. 186, p = 0.001∗∗), particularly in non-recent cases (p = 0.006∗∗). The COVID-19 group exhibited higher mean V'Epeak [% predicted] (123 vs. 115, p = 0.008∗∗) and fewer abnormal values (68 % vs. 79 %, p = 0.008∗∗), with significant improvements in the non-recent subgroup (p = 0.010∗∗). Other cardiopulmonary exercise testing measurements remained consistent across both groups (Table 3).

**Table 3:** Comparison of Cardiopulmonary Exercise Testing (CPET) and Pulmonary Function Outcomes (Simple Mean Differences) This table presents the unadjusted differences in CPET and pulmonary function outcomes between the control and COVID-19 groups. Further subgroup analysis is included for asymptomatic, recent, and non-recent COVID-19 cases.table_3 docx

				COVID-19 SUBGROUPS					
Outcomes	Control	COVID-19	p value	COVID-19 (asymptomatic)	p value	COVID-19 (<6 months)	p value	COVID-19 (>6 months)	p value
**CPET**									
Aerobic/anaerobic response									
*V'O2peak [% predicted]*	97 (87, 106)	96 (89, 105)	0.732	92 (86, 101)	0.312	98 (90, 109)	0.988	97 (89, 105)	0.980
*Abnormal value (* < *85 %)*	53 (22 %)	35 (15 %)	**0.045∗**	11 (24 %)	0.994	2 (11 %)	0.582	22 (13 %)	0.079
*V'O2 @ AT [% predicted V'O2peak]*	39 (34, 44)	38 (34, 42)	0.144	37 (32, 41)	0.169	40 (36, 46)	>0.999	38 (34, 43)	0.162
*Abnormal value (* < *40 %)*	122 (51 %)	140 (60 %)	0.079	30 (65 %)	0.316	9 (47 %)	0.986	101 (59 %)	0.385
*WR @ Peak Exercise [% predicted]*	87 (76, 98)	84 (77, 93)	0.061	83 (75, 91)	0.094	86 (76, 98)	0.944	85 (78, 93)	0.295
*Abnormal value (* < *80 %)*	78 (33 %)	85 (36 %)	0.441	19 (41 %)	0.682	8 (42 %)	0.844	58 (34 %)	0.992
*WR @ AT [% predicted WR @ Peak Exercise]*	28 (24, 35)	27 (24, 32)	**0.040∗**	26 (24, 31)	0.139	30 (24, 33)	0.885	27 (24, 32)	**0.026∗**
*Cutoff undefined*	–	–	–	–	–	–	–	–	–
Cardiovascular response									
*V′O2/HR @ Peak Exercise [% predicted]*	95 (84, 105)	93 (85, 103)	0.299	92 (85, 102)	0.210	97 (88, 110)	0.965	93 (85, 103)	0.103
*Cutoff undefined*	–	–	–	–	–	–	–	–	–
*V′O2/HR @ AT [% predicted V′O2/HRpeak]*	59 (52, 69)	58 (51, 64)	**0.023∗**	57 (49, 66)	0.347	59 (56, 65)	0.998	58 (51, 64)	**0.025∗**
*Cutoff undefined*	–	–	–	–	–	–	–	–	–
*HR @ Peak Exercise [% predicted]*	104 (100, 108)	105 (101, 108)	0.442	104 (101, 106)	0.975	103 (100, 106)	0.937	105 (102, 108)	0.298
*Abnormal value (* < *90 %)*	7 (2.9 %)	6 (2.6 %)	>0.999	2 (4.4 %)	0.943	0 (0 %)	0.876	4 (2.4 %)	0.984
*SBP @ Peak Exercise [mmHg]*	186 (169, 205)	178 (162, 196)	**0.001∗∗**	185 (167, 197)	0.824	172 (152, 182)	0.017∗	177 (163, 197)	**0.006∗∗**
*Abnormal value (* > *220)*	29 (12 %)	16 (7.2 %)	0.083	4 (9.3 %)	0.930	0 (0 %)	0.307	12 (7.5 %)	0.384
Ventilatory response									
*V'Epeak [% predicted]*	115 (100, 134)	123 (102, 142)	**0.008∗∗**	114 (98, 136)	0.879	130 (113, 146)	**0.826**	124 (107, 144)	**0.047∗**
*Cutoff undefined*	–	–	–	–	–	–	–	–	–
*V'Epeak [% MVV]*	76 (66, 83)	78 (68, 88)	0.088	73 (64, 84)	0.960	77 (65, 89)	0.990	79 (69, 89)	0.086
*Abnormal value (* < *85 %)*	188 (79 %)	156 (68 %)	**0.008∗∗**	35 (80 %)	>0.999	13 (68 %)	0.720	108 (65 %)	**0.010∗∗**
Gas exchange response									
*SpO2 Desaturation [%]*	2.0 (0.9, 4.3)	1.5 (0.6, 3.5)	0.078	1.6 (0.8, 3.5)	>0.999	1.8 (0.8, 3.8)	0.993	1.4 (0.6, 3.4)	0.734
*Abnormal value (* > *5 %)*	46 (20 %)	35 (15 %)	0.223	7 (16 %)	0.907	4 (21 %)	0.999	24 (15 %)	0.538
*V′E/V′O2 [unit]*	51 (46, 56)	51 (46, 56)	0.858	51 (46, 54)	0.891	52 (46, 60)	0.970	51 (47, 56)	0.971
*Cutoff undefined*	–	–	–	–	–	–	–	–	–
*V′E/V′CO2 [unit]*	39.3 (36.3, 43.0)	39.3 (36.7, 42.7)	0.580	38.6 (35.7, 42.8)	0.950	40.5 (37.3, 42.4)	0.984	39.3 (37.0, 42.7)	>0.999
*Cutoff undefined*	–	–	–	–	–	–	–	–	–
Metabolic response									
*RER [unit] @ Peak Exercise*	1.24 (1.20, 1.29)	1.23 (1.18, 1.28)	0.074	1.23 (1.19, 1.29)	0.792	1.20 (1.15, 1.25)	0.204	1.23 (1.18, 1.28)	0.763
*Abnormal value (* < *1.15)*	20 (8.6 %)	28 (12 %)	0.223	7 (15 %)	0.532	5 (26 %)	0.071	16 (9.9 %)	0.976
**Spirometry**									
*FEV1/FVC [% predicted] (Tiffeneau Index)*	106 (100, 114)	105 (100, 112)	0.308	107 (102, 113)	0.999	103 (100, 113)	0.960	104 (99, 111)	0.815
*Abnormal value (* < *88 %)*	8 (3.4 %)	9 (3.9 %)	0.810	1 (2.3 %)	0.985	1 (5.9 %)	0.955	7 (4.1 %)	0.986
*FVC [% VC] (Air Trapping Dynamic)*	105.6 (103.4, 108.2)	105.0 (103.3, 107.4)	0.213	105.6 (103.8, 107.9)	>0.999	105.3 (103.4, 106.1)	0.966	104.8 (103.2, 107.1)	0.993
*Abnormal value (* < *90 %)*	0 (0 %)	0 (0 %)		0 (0 %)		0 (0 %)		0 (0 %)	
*RV/TLC [% predicted] (Air Trapping Static)*	56 (43, 69)	58 (46, 72)	0.182	58 (48, 69)	0.497	57 (47, 72)	0.967	59 (45, 72)	0.625
*Abnormal value (* > *130 %)*	211 (100 %)	208 (100 %)	0.498	42 (98 %)	**0.022∗**	17 (100 %)	>0.999	149 (100 %)	>0.999
*FRC [% predicted] (Hyperinflation)*	94 (83, 106)	95 (80, 107)	0.977	100 (83, 108)	0.788	101 (91, 112)	0.344	93 (80, 105)	0.869
*Abnormal value (* > *130 %)*	209 (99 %)	203 (97 %)	0.174	42 (98 %)	0.932	16 (94 %)	0.481	145 (97 %)	0.636
*MMEF [% predicted] (Small Airways)*	99 (82, 114)	96 (83, 111)	0.378	99 (85, 111)	0.989	89 (79, 100)	0.550	96 (81, 111)	0.885
*Abnormal value (* < *60 %)*	6 (2.6 %)	9 (3.9 %)	0.445	2 (4.7 %)	0.895	0 (0 %)	0.939	7 (4.1 %)	0.830
**Diffusion Capacity**									
*TLC [% predicted]*	89 (85, 94)	89 (84, 94)	0.384	90 (84, 96)	0.997	91 (88, 95)	0.895	88 (84, 94)	0.683
*Abnormal value (* < *80 %)*	21 (10.0 %)	21 (10 %)	>0.999	4 (9.3 %)	>0.999	1 (5.9 %)	0.950	16 (11 %)	0.995
*DLCO [% predicted]*	107 (100, 116)	104 (97, 113)	**0.028∗**	102 (96, 112)	0.427	102 (99, 112)	0.979	105 (97, 113)	0.204
*Abnormal value (* < *75 %)*	2 (0.9 %)	1 (0.5 %)	>0.999	0 (0 %)	0.908	0 (0 %)	0.971	1 (0.7 %)	0.990
*KCO [% predicted]*	115 (108, 123)	113 (106, 124)	0.399	114 (107, 120)	0.764	110 (106, 117)	0.537	114 (108, 125)	0.951
*Abnormal value (* < *75 %)*	0 (0 %)	2 (1.0 %)	0.247	0 (0 %)	>0.999	0 (0 %)	>0.999	2 (1.3 %)	0.265
**Breathing Monitor**									
*FeNO [ppb]*	22 (14, 33)	19 (13, 28)	**0.035∗**	18 (13, 28)	0.521	16 (11, 56)	**0.013∗**	19 (14, 26)	0.438
*Abnormal value (* > 50 ppb*)*	29 (13 %)	20 (8.4 %)	0.130	2 (4.4 %)	0.310	5 (26 %)	0.276	13 (7.5 %)	0.275

In pulmonary function and inflammation analysis, DLCO was slightly lower in the COVID-19 group compared to the control group (104 vs. 107, p = 0.028∗). FeNO levels were also lower in the COVID-19 group (19 ppb vs. 22 ppb, p = 0.035∗), with a significant reduction in recent COVID-19 cases (16 ppb, p = 0.013∗). Spirometry results, including FEV1/FVC ratios, showed no significant differences (Table 3).

### 5.4 Propensity score-weighted analysis

The propensity score-weighted analysis showed no significant differences in most CPET cardiopulmonary variables between the control and COVID-19 groups or within subgroup analyses. No observed differences were found in anaerobic and aerobic responses, gas exchange responses, and metabolic responses (Table 4).

**Table 4:** Propensity Score-Weighted Analysis of Cardiopulmonary Exercise Testing (CPET) and Pulmonary Function Outcomes This table shows the propensity score-weighted analysis of CPET and pulmonary function outcomes between the control and COVID-19 groups. Additional subgroup analysis is provided for asymptomatic, recent, and non-recent COVID-19 cases.table_4 docx

				COVID-19 SUBGROUPS					
Outcomes	Control	COVID-19	p value	COVID-19 (asymptomatic)	p value	COVID-19 (<6 months)	p value	COVID-19 (>6 months)	p value
**CPET**									
Aerobic/anaerobic response									
*V'O2peak [L/min]*	–	−0.28 (95 % CI -2.26, 1.70)	0.783	−1.64 (95 % CI -5.43, 2.14)	0.394	1.15 (95 % CI -4.88, 7.17)	0.709	−0.24 (95 % CI -2.59, 2.10)	0.838
*V'O2 @ AT [L/min]*	–	−0.28 (95 % CI -2.26, 1.70)	0.783	−1.64 (95 % CI -5.43, 2.14)	0.394	1.15 (95 % CI -4.88, 7.17)	0.709	−0.24 (95 % CI -2.59, 2.10)	0.838
*WR @ Peak Exercise [W]*	–	−2.05 (95 % CI -7.75, 3.64)	0.480	−8.37 (95 % CI -19.18, 2.45)	0.129	3.94 (95 % CI -13.58, 21.46)	0.659	−0.93 (95 % CI -7.41, 5.55)	0.778
*WR @ AT [W]*	–	−2.05 (95 % CI -7.75, 3.64)	0.480	−8.37 (95 % CI -19.18, 2.45)	0.129	3.94 (95 % CI -13.58, 21.46)	0.659	−0.93 (95 % CI -7.41, 5.55)	0.778
Cardiovascular response									
*V'O2/HR @ Peak Exercise [ml]*	–	−1.50 (95 % CI -3.47, 0.47)	0.135	−2.95 (95 % CI -6.57, 0.67)	0.110	0.82 (95 % CI -4.78, 6.42)	0.774	−1.43 (95 % CI -3.60, 0.74)	0.197
*V'O2/HR @ AT [ml]*	–	−1.50 (95 % CI -3.47, 0.47)	0.135	−2.95 (95 % CI -6.57, 0.67)	0.110	0.82 (95 % CI -4.78, 6.42)	0.774	−1.43 (95 % CI -3.60, 0.74)	0.197
*HR @ Peak Exercise [bpm]*	–	1.19 (95 % CI -1.24, 3.63)	0.337	0.68 (95 % CI -4.20, 5.56)	0.785	−0.06 (95 % CI -7.28, 7.16)	0.987	1.38 (95 % CI -1.37, 4.12)	0.326
*SBP @ Peak Exercise [mmHg]*	–	−7.68 (95 % CI -12.14, −3.21)	**0.001∗∗**	0.96 (95 % CI -7.08, 9.01)	0.812	−14.60 (95 % CI -23.74, −5.46)	**0.002∗∗**	−9.07 (95 % CI -13.93, −4.22)	< **0.001∗∗∗**
Ventilatory response									
*V'Epeak [L/min]*	–	1.56 (95 % CI -1.12, 4.24)	0.254	−3.18 (95 % CI -7.98, 1.63)	0.195	3.42 (95 % CI -4.29, 11.13)	0.385	2.38 (95 % CI -0.65, 5.42)	0.124
*V'Epeak [% MVV]*	–	2.92 (95 % CI 0.32, 5.53)	**0.028∗**	−3.46 (95 % CI -7.58, 0.66)	0.099	−0.26 (95 % CI -8.15, 7.63)	0.948	4.41 (95 % CI 1.51, 7.31)	**0.003∗∗**
Gas exchange response									
*SpO2 Desaturation [%]*	–	−0.20 (95 % CI -0.82, 0.41)	0.514	−0.48 (95 % CI -1.59, 0.63)	0.393	0.12 (95 % CI -1.92, 2.16)	0.908	−0.28 (95 % CI -0.96, 0.40)	0.411
*V'E/V'O2 @ Peak Exercise [unit]*	–	−0.86 (95 % CI -5.23, 3.52)	0.698	−2.38 (95 % CI -7.47, 2.71)	0.354	−2.79 (95 % CI -9.24, 3.66)	0.388	−0.28 (95 % CI -5.98, 5.42)	0.923
*V'E/V'CO2 @ Peak Exercise [unit]*	–	−1.22 (95 % CI -26.61, 24.17)	0.923	−4.48 (95 % CI -32.54, 23.58)	0.747	−5.60 (95 % CI -32.48, 21.27)	0.678	9.46 (95 % CI -21.41, 40.32)	0.544
Metabolic response									
*RER [unit] @ Peak Exercise*	–	−0.00 (95 % CI -0.02, 0.01)	0.513	−0.02 (95 % CI -0.04, 0.01)	0.141	−0.04 (95 % CI -0.09, 0.01)	0.088	−0.00 (95 % CI -0.02, 0.01)	0.804
**Spirometry**									
*FEV1 [% VC] (Tiffeneau Index)*	–	−0.58 (95 % CI -2.13, 0.97)	0.462	1.96 (95 % CI -0.95, 4.87)	0.186	−3.22 (95 % CI -6.52, 0.07)	0.055	−0.83 (95 % CI -2.56, 0.90)	0.345
*FVC [% VC] (Air Trapping Dynamic)*	–	0.04 (95 % CI -0.82, 0.89)	0.936	0.26 (95 % CI -0.95, 1.48)	0.667	−1.05 (95 % CI -2.84, 0.74)	0.248	−0.03 (95 % CI -0.99, 0.93)	0.957
*RV [% TLC] (Air Trapping Static)*	–	0.45 (95 % CI -0.51, 1.41)	0.361	1.88 (95 % CI -0.95, 4.71)	0.192	−0.73 (95 % CI -3.70, 2.23)	0.627	0.40 (95 % CI -0.64, 1.44)	0.451
*FRC [L] (Hyperinflation)*	–	0.03 (95 % CI -0.11, 0.17)	0.641	0.11 (95 % CI -0.12, 0.33)	0.353	0.25 (95 % CI -0.06, 0.55)	0.118	−0.01 (95 % CI -0.17, 0.15)	0.885
*MMEF [L/s] (Small Airways)*	–	−0.10 (95 % CI -0.32, 0.11)	0.346	0.16 (95 % CI -0.27, 0.60)	0.461	−0.22 (95 % CI -0.63, 0.20)	0.302	−0.12 (95 % CI -0.37, 0.13)	0.332
**Diffusion Capacity**									
*TLC [L]*	–	−0.02 (95 % CI -0.20, 0.17)	0.867	−0.13 (95 % CI -0.39, 0.12)	0.303	0.31 (95 % CI -0.16, 0.77)	0.196	−0.02 (95 % CI -0.24, 0.20)	0.881
*DLCO [mmol/min/kPa]*	–	−0.17 (95 % CI -0.53, 0.19)	0.359	−0.43 (95 % CI -0.93, 0.07)	0.089	0.06 (95 % CI -0.55, 0.68)	0.838	−0.12 (95 % CI -0.54, 0.30)	0.570
*KCO [mmol/min/kPa/L]*	–	−0.03 (95 % CI -0.07, 0.02)	0.242	−0.03 (95 % CI -0.09, 0.04)	0.449	−0.07 (95 % CI -0.17, 0.03)	0.160	−0.02 (95 % CI -0.07, 0.03)	0.518
**Breathing Monitor**									
*FeNO [ppb]*	–	−1.01 (95 % CI -5.81, 3.79)	0.680	−4.17 (95 % CI -9.55, 1.21)	0.128	36.61 (95 % CI -13.00, 86.21)	0.148	−2.00 (95 % CI -7.23, 3.23)	0.453

Cardiovascular responses indicated a decrease in systolic blood pressure (SBP) at peak exercise in the COVID-19 group, with an average reduction of 7.68 mmHg compared to controls (p = 0.001∗∗). This reduction was more pronounced in recent COVID-19 cases (<6 months), with a decrease of 14.60 mmHg (p = 0.002∗∗), and persisted in the non-recent subgroup (>6 months) with a decrease of 9.07 mmHg (p = <0.001∗). No reduction was observed in the asymptomatic subgroup (Table 4).

Ventilatory responses showed an increase in V'Epeak [% predicted MVV] by2.92 % in the COVID-19 group, notably in the non-recent subgroup (p = 0.028∗∗). No significant changes were observed in peak ventilatory efficiency measures in other subgroups. Spirometry results, diffusion capacity and FeNO measurements showed no significant differences (Table 4).

## 6 Sensitivity analysis

**Fig. 1:**
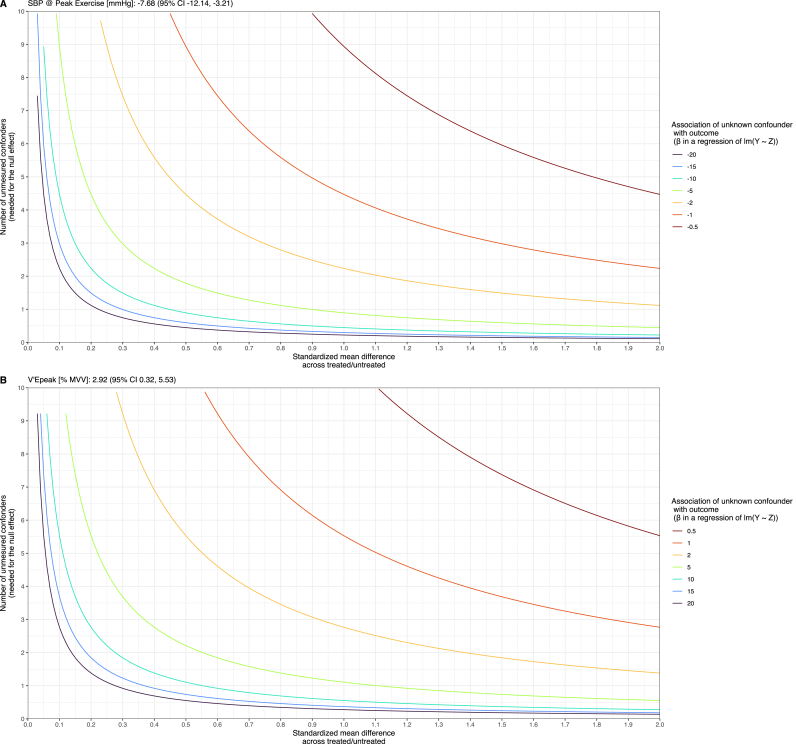
Sensitivity Analysis for Unmeasured Confounding This figure illustrates the sensitivity analysis for the impact of unmeasured confounding on systolic blood pressure (SBP) at peak exercise (−7.19 mmHg) and peak minute ventilation (V'Epeak%MVV; 3.33 %). The analysis includes the standardized mean difference across groups and the number of unmeasured confounders needed to nullify the observed effect.Fig. 1 pdf.

Sensitivity analysis for systolic blood pressure (SBP) at peak exercise revealed that under a weak negative association of −0.5, approximately 30, 18, and 11 unmeasured confounders are needed to nullify the observed effects for small (SMD = 0.30), medium (SMD = 0.50), and large (SMD = 0.80) effect sizes, respectively. With a moderate negative association of −5, the number of necessary confounders is about 3 for small, less than 2 for medium, and 1 for large effect sizes. At a strong negative association of −20, fewer than one confounder is needed across all effect sizes (Fig. 1A).

For V'Epeak [% predicted MVV], under a weak positive association of +0.5, approximately 36, 22, and 13 unmeasured confounders are necessary for small, medium, and large effect sizes, respectively. With a moderate positive association of +5, around 3 for small, 2 for medium, and 1 for large effect sizes are required. At a strong positive association of +20, less than one confounder is needed across all effect sizes (Fig. 1B).

## 7 Discussion

This study shows significant and cardiopulmonary effects, lasting at least 6 months, in young adults post-COVID-19. Key findings include a significant reduction in systolic blood pressure at peak exercise, with an average decrease of 7.68 mmHg in the COVID-19 group compared to controls, particularly pronounced in recent symptomatic cases. Additionally, the COVID-19 group showed a 2.92 % increase in peak ventilatory response (V'Epeak [% predicted MVV]), especially in the subgroup exposed more than 6 months prior to testing. These results emphasize the persistent impact of COVID-19 on cardiovascular and ventilatory function. While other CPET variables, spirometry results, and pulmonary tests showed no significant differences, the observed changes in systolic blood pressure and ventilatory response highlight the persisting physiological consequences of COVID-19, even in a young and predominantly healthy population.

The significant reduction in systolic blood pressure (SBP) observed in our young adults post-COVID-19 cohort aligns with findings from other studies investigating cardiovascular sequelae of COVID-19. For instance, a study from the Mayo Clinic exercise testing database found that low SBP and other autonomic abnormalities were more frequently observed during cardiopulmonary exercise testing (CPET) in long COVID patients compared to controls (34 % vs. 23 %, P < 0.04), even after adjusting for age, sex, beta blocker use, and effort based on respiratory exchange ratio [22]. Additionally, a study examining Arizona firefighters found significantly lower SBP and diastolic blood pressure (DBP) at peak exercise during post-COVID CPET annual examinations [23]. Chan et al. proposed that a previous symptomatic SARS-CoV-2 infection could modify blood pressure regulation during exercise in healthy individuals by affecting the autonomic nervous system [24]. The persistence of these disturbances for over six months, as found in our results, may indicate persisting cardiovascular sequelae in young adults, particularly cardiovascular autonomic dysfunction (CVAD), a common feature of long COVID [25,26].

Our findings are consistent with various studies on post-viral syndromes and CVAD, which have documented cardiovascular autonomic dysfunctions, including orthostatic hypotension, postural orthostatic tachycardia syndrome (POTS), and inappropriate sinus tachycardia [[27], [28], [29], [30]]. These conditions suggest that COVID-19 may impair autonomic control, potentially through viral invasion of the central nervous system and subsequent neurotropism affecting the cranial nerves [31,32]. The involvement of angiotensin-converting enzyme 2 (ACE2) receptors, highly expressed in neural structures, further supports this mechanism [33]. Additionally, low-grade inflammation and impaired baroreflex function may contribute to the observed autonomic abnormalities, including reduced SBP during exercise [34].

We did not find any lung impairment in our results comparing exposed and non-exposed groups, either in diffusion capacity or spirometry, contrary to what has been observed in other studies. This discrepancy could be due to our cohort primarily consisting of young previously healthy adults with mild infections and therefore limited sequelae. The main explanation for the observed 2.92 % increase in peak ventilatory response is likely deconditioning and muscle weakness. This finding aligns with other research indicating that deconditioning is a major factor in impaired exercise response among COVID-19 survivors. A study by Mancini et al. (2021) found that COVID-19 survivors exhibited significant reductions in exercise capacity and muscle strength, attributing these changes to prolonged periods of inactivity during recovery [35]. Similarly, a study by Barizien et al. (2021) reported that deconditioning, rather than pulmonary impairment, was the primary contributor to exercise intolerance in post-COVID-19 patients [36]. Deconditioning, resulting from prolonged inactivity during and after illness, leads to reduced muscle strength and a decline in overall physical fitness, necessitating a higher ventilatory response to meet oxygen demands during exercise.

Additionally, our findings in the main study indicate significant trends toward metabolic disorders in the non-recent COVID-19 group compared to the control group. These trends include higher BMI (24.0 kg/m^2^ vs. 23.2 kg/m^2^; p = 0.035), lower aerobic threshold (39 % vs. 41 %; p = 0.012), and elevated blood cholesterol (4.2 μM vs. 3.9 μM; p < 0.0001) and LDL concentrations (2.4 μM vs. 2.2 μM; p = 0.001). The only notable psychosocial difference was higher fatigue scores in the non-recent COVID-19 group (median 12 points vs. 11; p = 0.027) [8]. These findings are supported by other studies, such as Huang et al. (2021), which demonstrated long-term metabolic and cardiovascular changes in post-COVID-19 patients [28], and Carfi et al. (2020), which reported persistent symptoms, including fatigue and metabolic disturbances, several months after the acute infection [37]. Therefore, the increased ventilatory response and reduced exercise capacity observed in our study are likely attributable to deconditioning and muscle weakness. Increased fatigue, although not directly linked to stress and anxiety in our findings, likely contributes to further deconditioning, exacerbating the ventilatory response.

### 7.1 Limitations and strengths

This study has several strengths, including its focus on a young cohort with few comorbidities, which reduces confounding factors related to pre-existing health conditions. Additionally, the use of propensity score weighting allowed us to adjust for potential confounders and better estimate causal differences between the COVID-19 and control groups. This methodological rigor enhances the validity of our findings regarding persisting cardiopulmonary sequelae of COVID-19.

However, there are also important limitations to consider. First, the observed differences in cardiopulmonary responses do not indicate pathological values, and further research is needed to determine the clinical significance of these findings. Second, our cohort was not restricted to individuals with persistent symptoms and therefore does not represent a classical long COVID population; however, our findings may reflect subclinical or early post-acute sequelae in young adults. Third, some measurements, such as maximum voluntary ventilation (MVV), were estimated based on formulas rather than directly measured. This could introduce errors and affect the accuracy of our results. Fourth, the low proportion of female participants (5.4 % in the control group and 6.6 % in the COVID-19 group) limits the generalizability of our findings to both sexes. Lastly, while our study focused on a specific age group and population, the findings may not be applicable to older adults or those with different health profiles.

## 8 Implications and further research

Our study demonstrates that significant cardiopulmonary impacts can be observed post-COVID-19 even in young, previously healthy adults. This underscores the importance of maintaining physical activity during and after recovery to mitigate deconditioning and preserve cardiopulmonary function. Encouraging regular exercise, during recovery, could help prevent the muscle weakness and increased ventilatory response noted in our study.

Future research should focus on longitudinal studies to track the evolution of these cardiopulmonary effects over at least a year to determine whether symptoms attenuate or persist. Including a more balanced gender representation and diverse health profiles would enhance generalizability. Additionally, investigating the underlying mechanisms, such as autonomic dysfunction or inflammatory processes, could provide deeper insights and inform targeted rehabilitation strategies.

## 9 Conclusion

This study reveals significant and cardiopulmonary effects, persisting at least for 6 months, in young adults post-COVID-19, including a reduction in systolic blood pressure at peak exercise and an increase in peak ventilatory response. These findings, although not necessarily pathological, highlight the persistent impact of COVID-19 on cardiovascular and ventilatory function, even in a young and predominantly healthy population. While no significant lung impairment was detected, the observed changes are likely attributable to deconditioning and muscle weakness resulting from prolonged inactivity during and after illness. Our results underscore the importance of maintaining physical activity during recovery to mitigate deconditioning and preserve cardiopulmonary function. The study's strengths include a focus on a young cohort with minimal comorbidities and the use of propensity score weighting to adjust for confounding factors. However, limitations such as the low proportion of female participants and reliance on estimated measurements should be considered when interpreting the results. Further research is needed to track the long-term evolution of these effects, include a more balanced gender representation, and explore the underlying mechanisms driving these changes.

## CRediT authorship contribution statement

**Thibault Lovey:** Writing – review & editing, Writing – original draft, Visualization, Methodology, Investigation, Formal analysis, Conceptualization. **Nejla Gültekin:** Writing – review & editing, Funding acquisition. **Zeno Stanga:** Writing – review & editing. **Andreas Stettbacher:** Writing – review & editing, Funding acquisition. **Jeremy Werner Deuel:** Writing – review & editing, Methodology, Investigation, Funding acquisition, Data curation, Conceptualization. **Patricia Schlagenhauf:** Writing – review & editing, Writing – original draft, Validation, Supervision, Methodology, Investigation, Funding acquisition, Data curation, Conceptualization.

## Data sharing

Due to data protection regulations, individual data cannot be directly shared by the authors. However, we can provide details about the test battery, questionnaires used, and some deidentified aggregated data. For data sharing requests, please contact the Principal Investigator (patricia.schlagenhauf@uzh.ch) after the paper is published and a formal request for data sharing has been submitted. For transparency and reproducibility, the R code used for the analysis is available in Appendix A.

## Role of the funding source

This study was funded by the Swiss Armed Forces. The funder had no involvement in the study design, data collection, data analysis, or interpretation of the results.

## Declaration of competing interest

The authors declare that they have no known competing financial interests or personal relationships that could have appeared to influence the work reported in this paper.

## Appendix A

HTML Code for Reproducing Figures and Tables.

This appendix includes an HTML file containing the detailed R code used to create all the tables and figures featured in this article. To access the code, please download and open the HTML file in your preferred web browser. This appendix is provided to ensure transparency and reproducibility of the analyses performed in this study. locomoT.html.
